# S-adenosylmethionine and methylthioadenosine inhibit cancer metastasis by targeting microRNA 34a/b-methionine adenosyltransferase 2A/2B axis

**DOI:** 10.18632/oncotarget.20234

**Published:** 2017-08-12

**Authors:** Maria Lauda Tomasi, Carla Cossu, Ylenia Spissu, Andrea Floris, Minjung Ryoo, Ainhoa Iglesias-Ara, Qiang Wang, Stephen J. Pandol, Neil A. Bhowmick, Ekihiro Seki, Edwin M. Posadas, Shelly C. Lu

**Affiliations:** ^1^ Division of Digestive and Liver Diseases, Department of Medicine, Cedars-Sinai Medical Center, Los Angeles, California, USA; ^2^ Department of Medicine, University of Sassari, Sassari, Italy; ^3^ Department of Biomedical Science, University of Cagliari, Cagliari, Italy; ^4^ Department of Genetics, Faculty of Science and Technology, University of The Basque Country, Bilbao, Spain; ^5^ Department of Biomedical Sciences, Cedars-Sinai Medical Center, Los Angeles, California, USA; ^6^ Urologic Oncology Program, Division of Hematology & Oncology, Department of Medicine, Cedars-Sinai Medical Center, Los Angeles, California, USA; ^7^ Translational Oncology Program, Samuel Oschin Comprehensive Cancer Institute, Cedars-Sinai Medical Center, Los Angeles, California, USA; ^8^ Cancer Biology Program, Samuel Oschin Comprehensive Cancer Institute, Cedars-Sinai Medical Center, Los Angeles, California, USA

**Keywords:** S-adenosylmethionine, methylthioadenosine, miR-34 family, methionine adenosyltransferase genes, cancer metastasis

## Abstract

MicroRNA-34a (miR-34a) is down-regulated in colorectal cancers (CRC) and required for interleukin-6 (IL-6)-induced CRC metastasis. Mice lacking miR-34a developed more invasive cancer in a colitis-associated cancer model. In the same model, S-adenosylmethionine (SAMe) and methylthioadenosine (MTA) inhibited IL-6/STAT3 and lowered tumor burden. SAMe and MTA reduce the expression of methionine adenosyltransferase 2A (MAT2A) and there are consensus binding sites for miR-34a/b in the *MAT2A* 3’UTR. Here we examined whether SAMe/MTA influence miR-34a/b expression and cancer metastasis. We found SAMe and MTA raised miR-34a/b expression in CRC cell lines, inhibited migration and invasion *in vitro* and liver metastasis *in vivo*. Like CRC, MAT2A and MAT2B expression is induced in human pancreas and prostate cancers. Treatment with SAMe, MTA, miR-34a or miR-34b inhibited MAT2A expression mainly at the protein level. MAT2B protein level also fell because MAT2A and MAT2B enhance each other’s protein stability. Overexpressing miR-34a or miR-34b inhibited while MAT2A or MAT2B enhanced CRC migration and invasion. Co-expressing either miR-34a/b had minimal to no effect on MAT2A/MAT2B’s ability to increase migration, invasion and growth. Taken together, MAT2A and MAT2B are important targets of miR-34a/b and SAMe and MTA target this axis, suppressing MAT2A/MAT2B while raising miR-34a/b expression, inhibiting cancer metastasis.

## INTRODUCTION

MicroRNAs (miRNAs) are a class of small non-coding RNAs that regulate gene expression at the post-transcriptional level [1]. MiRNAs are deregulated in human cancers, with up-regulation of multiple oncogenic miRNAs and down-regulation of tumor suppressor miRNAs [1]. MiR-34a is one of the tumor suppressor miRNAs that is down-regulated in multiple human cancers, including colorectal cancer (CRC), pancreas and prostate [1–3]. MiR-34a is part of a family that includes miR-34b and miR-34c, with miR-34a having its own transcript while the other two share a common primary transcript [4]. MiR-34 family members are transcriptional targets of p53 and p53 was shown to inhibit CRC metastasis by inducing miR-34a [5].

MiR-34a is part of a feedback loop, where interleukin-6 (IL-6) activated Signal transducer and activator of transcription 3 (STAT3) represses miR-34a expression and in return, miR-34a targets IL-6 receptor (IL-6R) to inhibit IL-6 signaling [5]. Mice lacking miR-34a developed more invasive cancer using a colitis-associated cancer model with azoxymethane/dextran sodium sulfate [5]. Using the same animal model, we reported S-adenosylmethionine (SAMe) and its metabolite methylthioadenosine (MTA) inhibited IL-6-STAT3 signaling and lowered tumor burden [6]. We have also shown SAMe and MTA lowered the expression of methionine adenosyltransferase 2A (MAT2A), which is overexpressed in human CRC [7].

MAT2A overexpression enhances cancer cell growth and survival, as there is a positive feedforward loop between MAT2A and polyamines [8] and MAT2A protein positively regulates B-Cell CLL/lymphoma 2 (BCL-2) expression [9]. There are consensus binding sites for miR-34a and miR-34b in the *MAT2A* 3’-UTR based on three different miRNA prediction target databases (TargetScan, mirDB, miRSVR, Segal Lab of Computational Biology). While miR-34a’s role in tumorigenesis has received a lot of attention, less is known about miR-34b. *MAT2B* encodes for a regulatory subunit that controls the activity of the *MAT2A*-encoded isoenzyme and is induced in parallel to MAT2A in CRC and liver cancer [10, 11]. The underlying mechanisms for MAT2A/MAT2B parallel induction in these cancers are not clear. The aims of the current work were to examine whether miR-34 family members regulate MAT2A and MAT2B expression and whether SAMe and MTA target this axis in multiple human cancers where miR-34a has been reported to be down-regulated.

## RESULTS

### Effects of SAMe and MTA on apoptosis, growth and migration in RKO and SW620 cells

**Figure 1:**
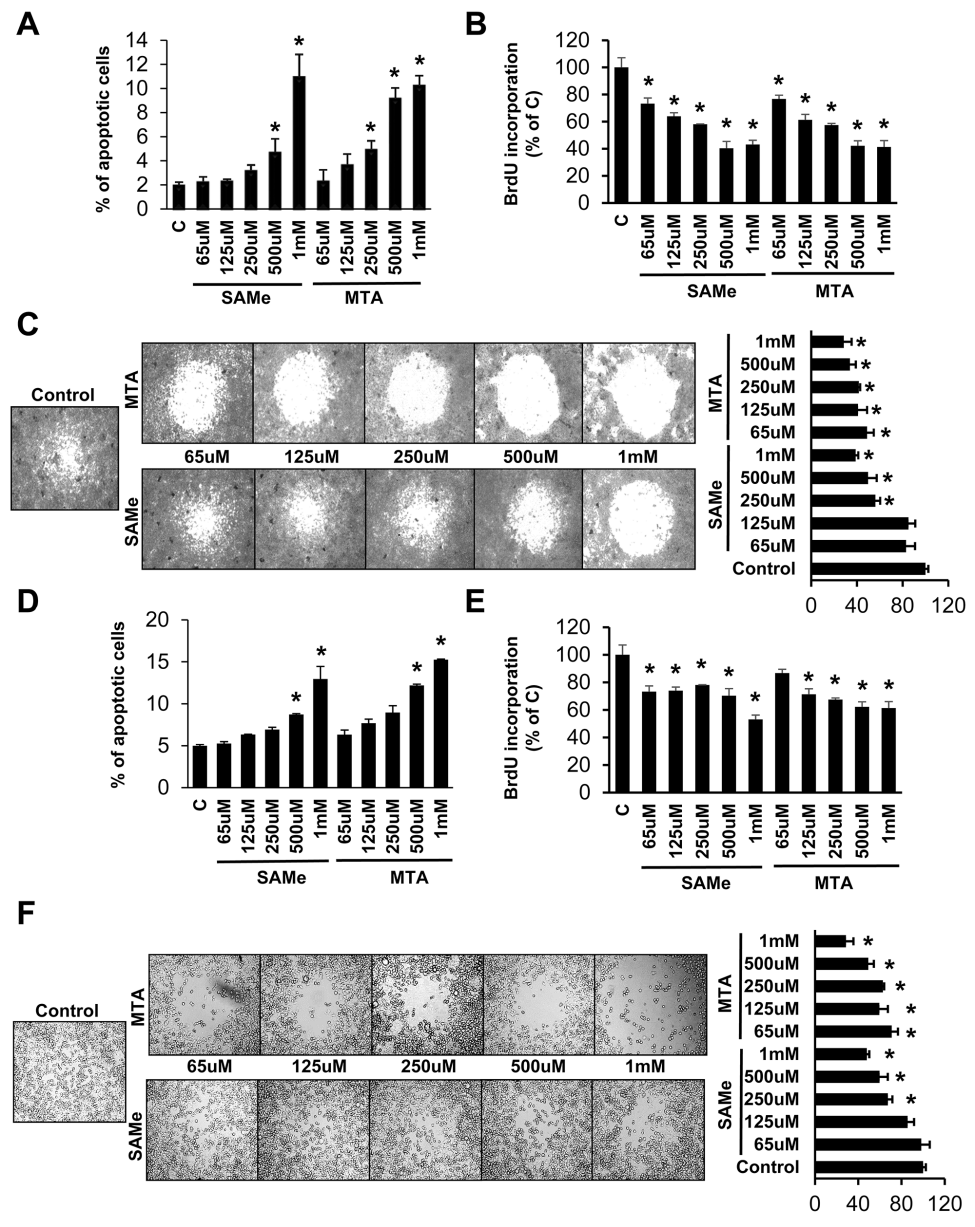
Effects of SAMe and MTA on apoptosis, growth and migration in RKO and SW620 cells RKO **(A-C)** and SW620 **(D-F)** cells were treated with varying concentrations of SAMe or MTA for 24 hours. Apoptosis, BrdU incorporation and cell migration were analyzed byas described in Methods. Results from 3-4 experiments are shown as mean % of apoptotic cells ± SEM and as % of control ± SEM for BrdU and migration, *p<0.04 vs control.

Since SAMe and MTA inhibited IL-6/STAT3 signaling in the colitis-associated colon cancer model [6] and this pathway was shown to be critical for cancer invasion and metastasis [5], we examined whether SAMe and MTA might inhibit cancer cell migration. We first defined the threshold dosage for SAMe and MTA’s pro-apoptotic and anti-proliferative effects in human CRC cell lines RKO and SW620. SAMe’s threshold dose for pro-apoptotic effect is 500 μM for both cell lines, whereas MTA’s threshold dose is lower in RKO cells at 250 μM (Figure 1A and 1D). Both agents exerted anti-proliferative effect at lower dosage (65-125 μM) as compared to apoptosis (Figure 1B and 1E). Effect of SAMe and MTA on cell migration was next examined and SAMe and MTA inhibited migration significantly at 250 μM and 65 μM, respectively, at doses below their pro-apoptotic threshold (Figure 1C and 1F).

### SAMe and MTA induce the expression of miR-34a and miR-34b and all four treatments lower the expression of MAT2A and MAT2B

**Figure 2:**
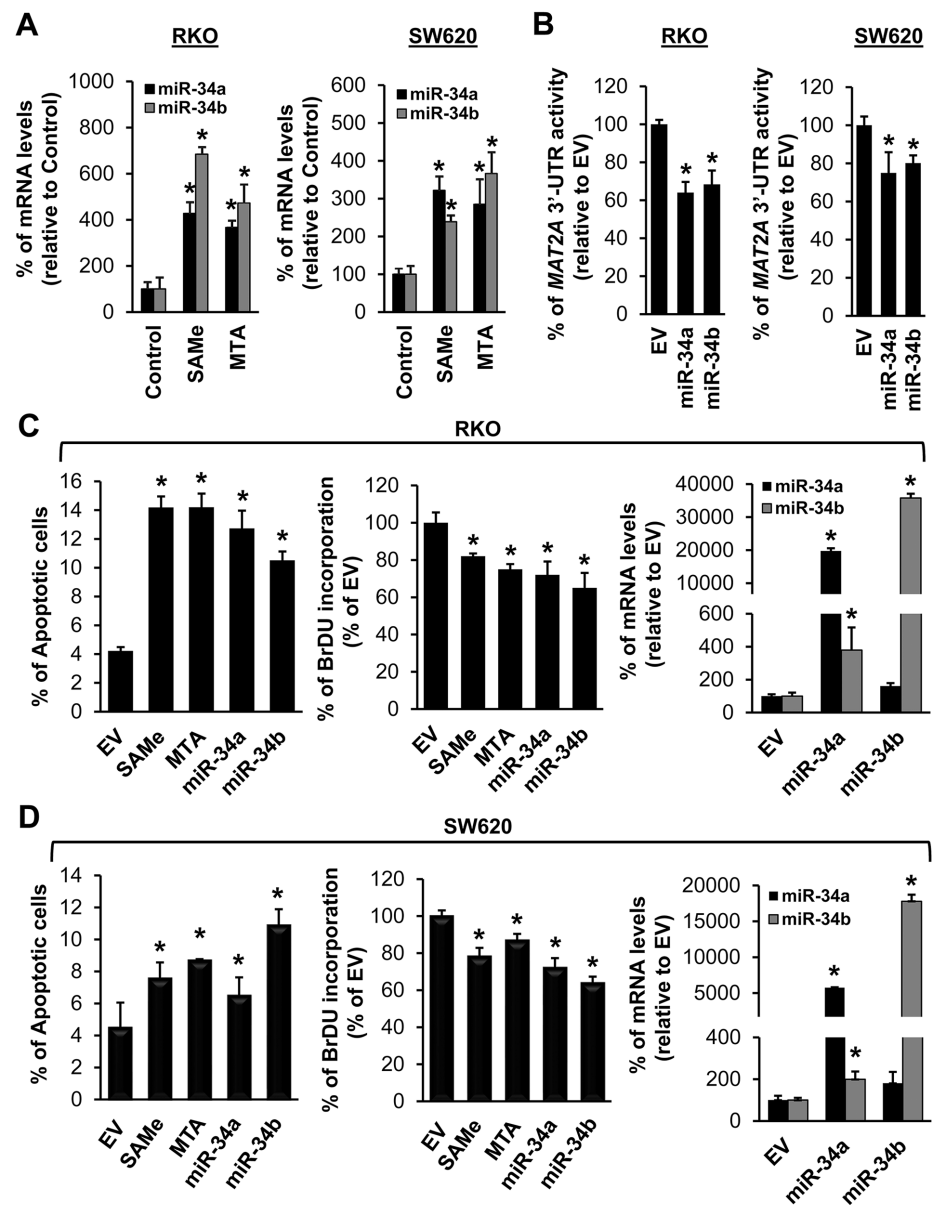
SAMe and MTA induce the expression of miR-34a and miR-34b and all four treatments induce apoptosis and inhibit growth of colon cancer cells **(A)** RKO (left) and SW620 (right) cells were treated with 250 μM SAMe or MTA for 24 hours. The mRNA levels of miR-34a and miR-34b were compared to vehicle treated controls using real-time PCR. Results represent mean ± SEM from 4-5 experiments, *p<0.001 vs. control. **(B)** *MAT2A* 3’UTR reporter activity in RKO and SW620 cells treated with miR-34a, miR-34b or empty vector (EV). Results represent mean ± SEM from 3 experiments, *p<0.01 vs. EV. RKO **(C)** and SW620 **(D)** cells were treated with 250 μM SAMe or MTA, overexpression of miR-34a or miR-34b as described in Methods for 24 hours and were processed for apoptosis, growth by BrdU, miR-34 and miR-34b transfection efficiency measurements. Results represent mean ± SEM from 3 experiments, *p<0.01 vs. EV.

**Figure 3:**
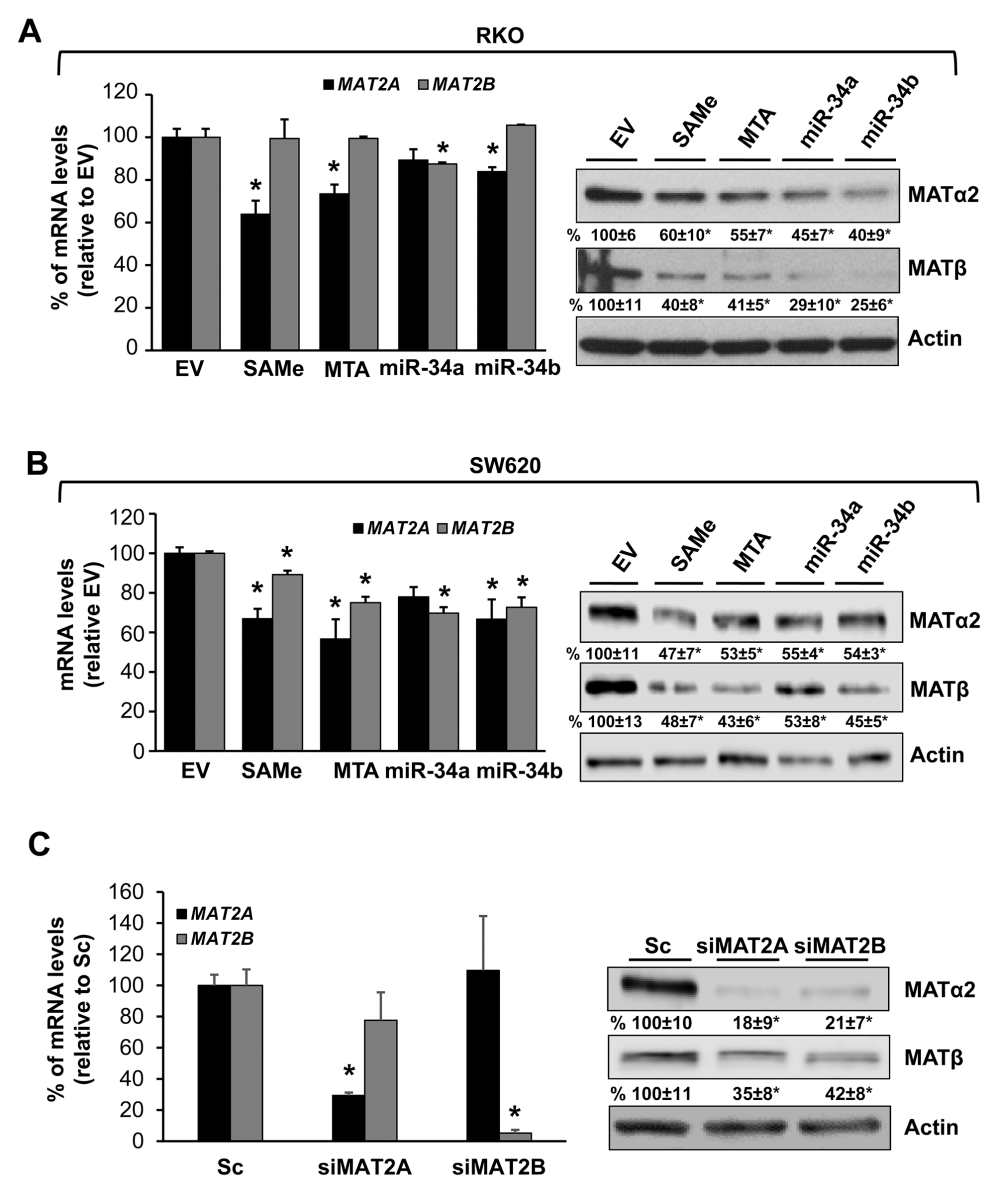
Effects of SAMe, MTA, miR-34a and miR-34b on MAT2A and MAT2B expression RKO **(A)** and SW620 **(B)** cells were treated with 250 μM SAMe or MTA, or overexpression of miR-34a or miR-34b as described in Methods for 24 hours. *MAT2A* mRNA levels, MAT2A and MAT2B protein levels (MATα2 and MATβ) were measured by real-time PCR and Western blotting, respectively. Densitometric changes are summarized below the blots. Results represent mean ± SEM from 3 to 4 experiments, *p<0.05 (real-time PCR) and *p<0.01 (Western blotting) vs. empty vector (EV) control. **(C)** RKO cells were treated with 10 nM siRNA against *MAT2A* or *MAT2B*, or scramble siRNA control (Sc) for 48 hours and MAT2A and MAT2B expression was measured by real-time PCR and Western blotting. Densitometric changes are summarized below the blots. Results represent mean ± SEM from 3 experiments, *p<0.001 vs. Sc.

We next examined whether SAMe and MTA treatment might influence miR-34a and miR-34b expression because they inhibit IL-6/STAT3 activation [6], which inhibits miR-34a expression [5], and they lower MAT2A expression [7], which contains possible binding sequences in its 3’UTR for miR-34a and miR-34b. SAMe and MTA treatment for 24 hours increased the mRNA levels of miR-34a and miR-34b in both CRC cell lines (Figure 2A) and a direct effect of miR-34a and miR-34b on *MAT2A* 3’UTR was confirmed using reporter assay (Figure 2B). When compared side by side, SAMe and MTA treatment for 24 hours exerted comparable effects on apoptosis and growth inhibition as overexpressing either miR-34a or miR-34b (Figure 2C and 2D). Treatment with SAMe or MTA lowered the *MAT2A* mRNA levels by about 35-45%, and the MAT2A-encoded protein (MATα2) levels by about 40-53% (Figure 3A and 3B). Overexpressing either miR-34a or miR-34b had minimal to no influence on *MAT2A* mRNA levels but they reduced MATα2 levels by 45-60% (Figure 3A and 3B). While the effects of all these treatments on *MAT2B* mRNA levels were minimal to none, all reduced MAT2B protein (MATβ) levels significantly (Figure 3A and 3B). MATβ is a regulatory subunit that interacts with MATα2 [13] and there is no consensus binding site in the 3’UTR of *MAT2B* for either miR-34a or miR-34b. We investigated whether the two proteins may stabilize each other. Consistently, knockdown of either *MAT2A* or *MAT2B* drastically reduced each other’s protein level (Figure 3C), supporting the notion that their interaction may help to stabilize each other at the protein level.

### Effects of MAT2A, MAT2B, miR-34a and miR-34b in colon cancer cell migration, invasion and growth

**Figure 4:**
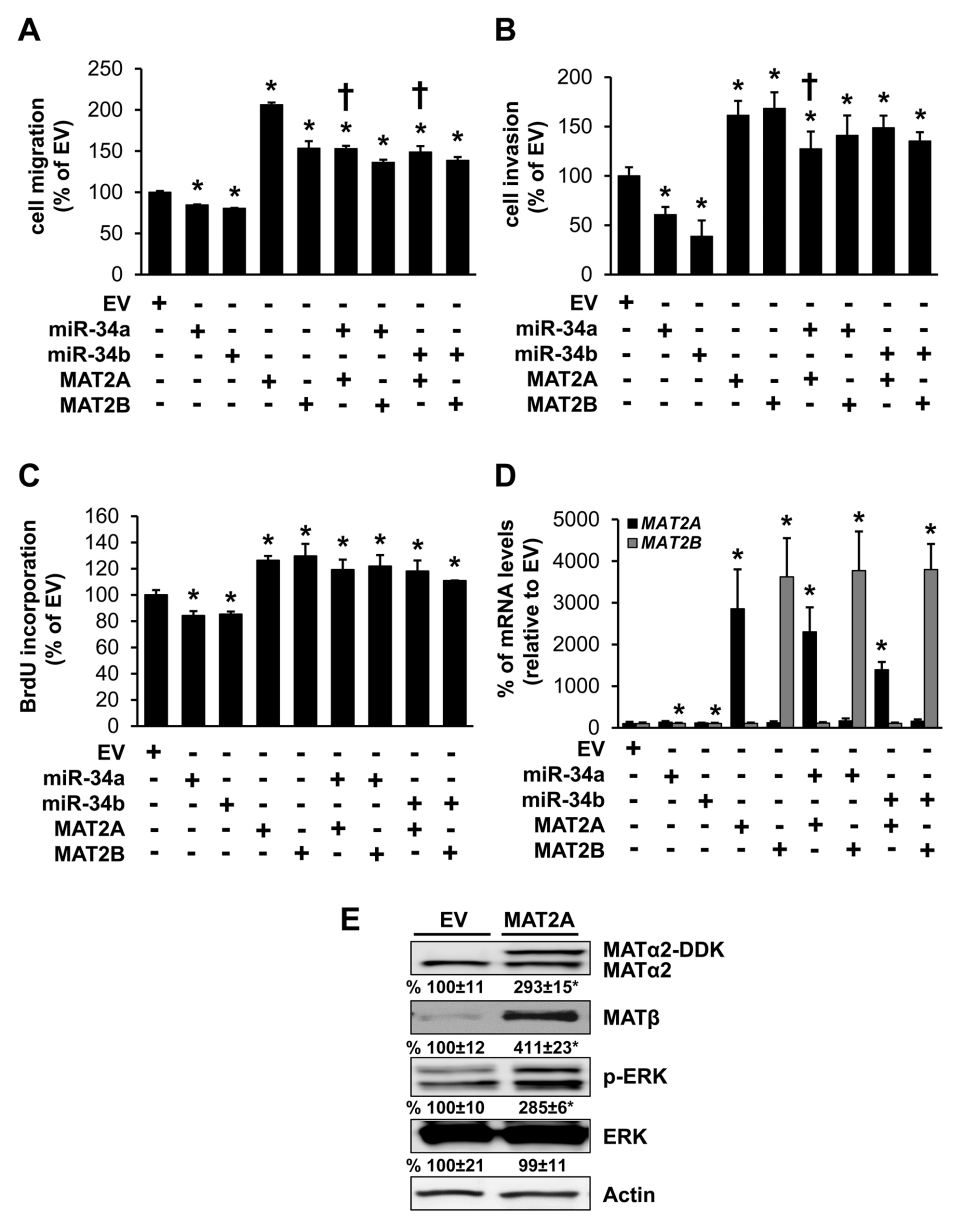
Effects of MAT2A, MAT2B, miR-34a and miR-34b in colon cancer cell migration, invasion and growth RKO cells were transfected with empty vector (EV), miR-34a, miR-34b, MAT2A or MAT2B expression vectors alone or in combination for 24 hours for measurement of cell migration **(A)**, invasion **(B)** and growth **(C)** as described in Methods. Results represent mean ± SEM from 3 to 4 experiments, *p<0.05 vs. EV. †p<0.05 vs. MAT2A overexpression. **(D)** shows transfection efficiency. **(E)** RKO cells were transfected with MAT2A overexpression vector in DDK tag for 24 hours and Western blotting was done for MATα2, MATβ, p-ERK, total ERK and Actin for housekeeping control. Densitometric values are summarized below the blots. Results represent mean ± SEM from 3 experiments, *p<0.04 vs. EV.

MAT2A expression is linked to increased growth and cancer cell survival [8, 9], while high MAT2B expression is linked to increased activity of RAS-RAF-MEK-ERK in colon cancer cells [11, 14]. We next investigated whether the effects of miR-34a and miR-34b on cancer cell migration, invasion and growth could be mediated in part by these two MAT proteins. Overexpressing miR-34a or miR-34b reduced RKO cell migration, invasion and growth; whereas overexpressing either MAT2A or MAT2B had the opposite effects (Figure 4A–4C). Interestingly, co-expressing miR-34a with MAT2A reduced slightly the inductive effect of MAT2A on cell migration and invasion but not growth. Co-expressing miR-34b with MAT2A only reduced the effect of MAT2A on migration slightly, but had no effect on invasion or growth. Co-expressing either miR-34a or miR-34b with MAT2B had no influence on MAT2B’s inductive effect on migration, invasion or growth (Figure 4A–4C). Transfection efficiency is shown in Figure 4D. MAT2A is not known to enhance cancer cell migration or invasion. However, if it enhances MAT2B expression, then it could activate MEK/ERK to drive cancer cell migration [15]. Consistently, overexpression of MAT2A (indicated by the DDK tag) increased endogenous MAT2B protein (MATβ) expression more than 3-fold and activated ERK (Figure 4E). Thus, miR-34a and miR-34b could impact MAT2B expression and its downstream signaling pathway indirectly via MAT2A.

### SAMe and MTA inhibit metastatic colon cancer growth in mice

**Figure 5:**
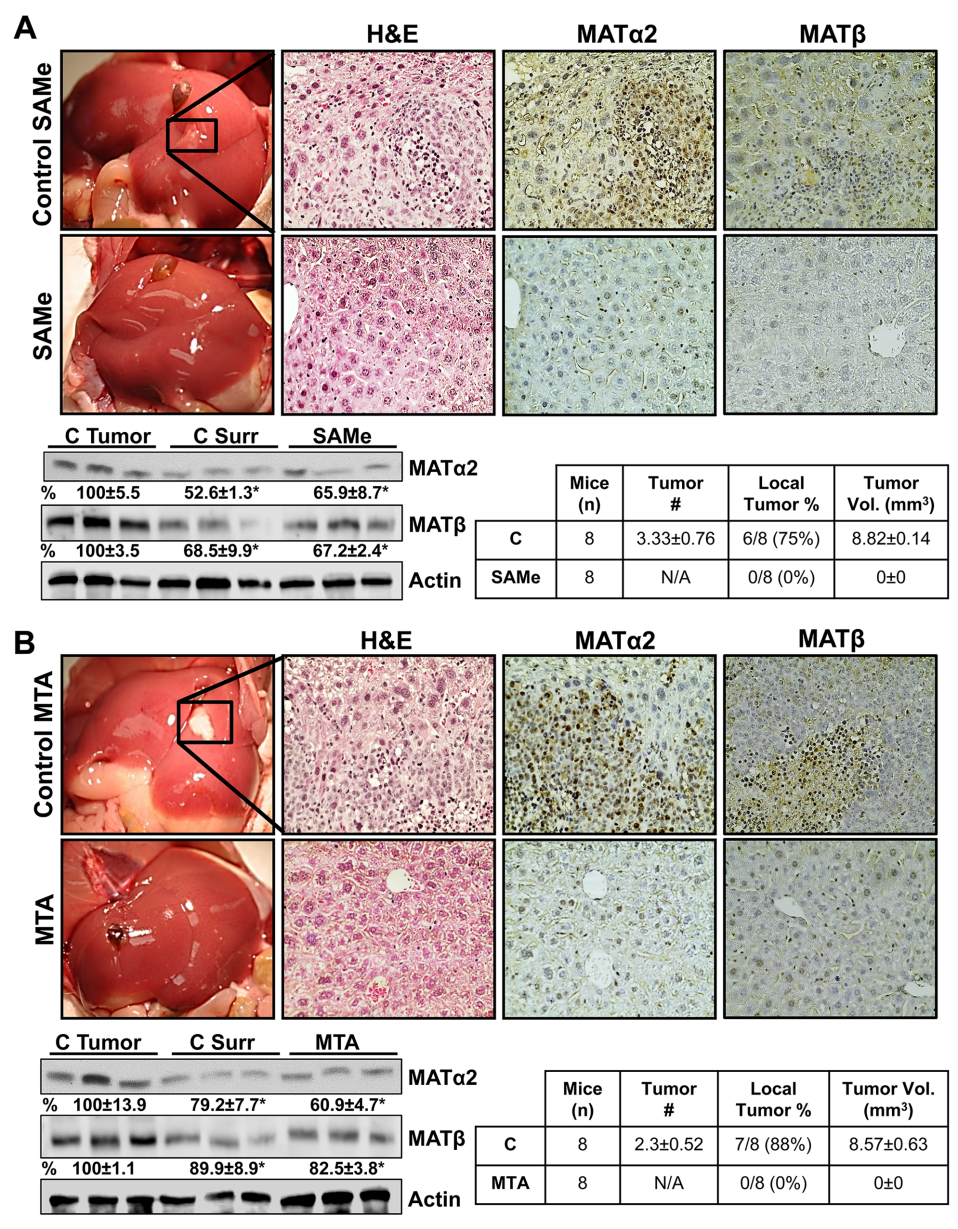
SAMe and MTA inhibit metastatic colon cancer growth in mice A metastatic CRC model was established as described in Methods. On day 4, mice were allocated to 4 groups (n=8 per group): 1. vehicle control for SAMe, 2. SAMe (100 mg/kg/day), 3. DMSO (vehicle for MTA), and 4. MTA (75 mg/kg/day). All mice were euthanized on day 21. **(A)** shows gross liver morphology, H&E and IHC for MATα2 and MATβ in the SAMe treated and control groups. **(B)** shows the same for the MTA and its control group (magnification 40x). SAMe and MTA treated groups did not have visible tumor, whereas both control groups had tumors in the majority. Some of the mice had one tumor easily visible, whereas others had multiple tumors. Tumor volume is an average of the tumors in each mouse. Representative Western blotting for MATα2 and MATβ are shown comparing their expression in the tumors, surrounding liver tissues, and SAMe or MTA treated livers from n=5 per group. Densitometric values are summarized below the blots, *p<0.03 vs. respective tumor controls.

Next, we examined whether SAMe and MTA treatment can inhibit colon cancer growth in a metastatic liver expansion model. Treatment with either SAMe or MTA completely abrogated any sign of CRC in the liver (Figure 5A and 5B) or any other organ (not shown). Liver protein levels of MATα2 and MATβ in SAMe or MTA treated mice are similar to the adjacent liver tissues that surrounded the tumors and are lower than the tumors from vehicle treated mice (Figure 5A and 5B). Mice tolerated the treatment regimen with SAMe or MTA well without any toxicity as measured by appearance, behavior, food and water intake as compared to their respective controls.

### MAT2A and MAT2B expression is upregulated in human prostate and pancreas cancers and down-regulated by SAMe, MTA, miR-34a and miR-34b

**Figure 6:**
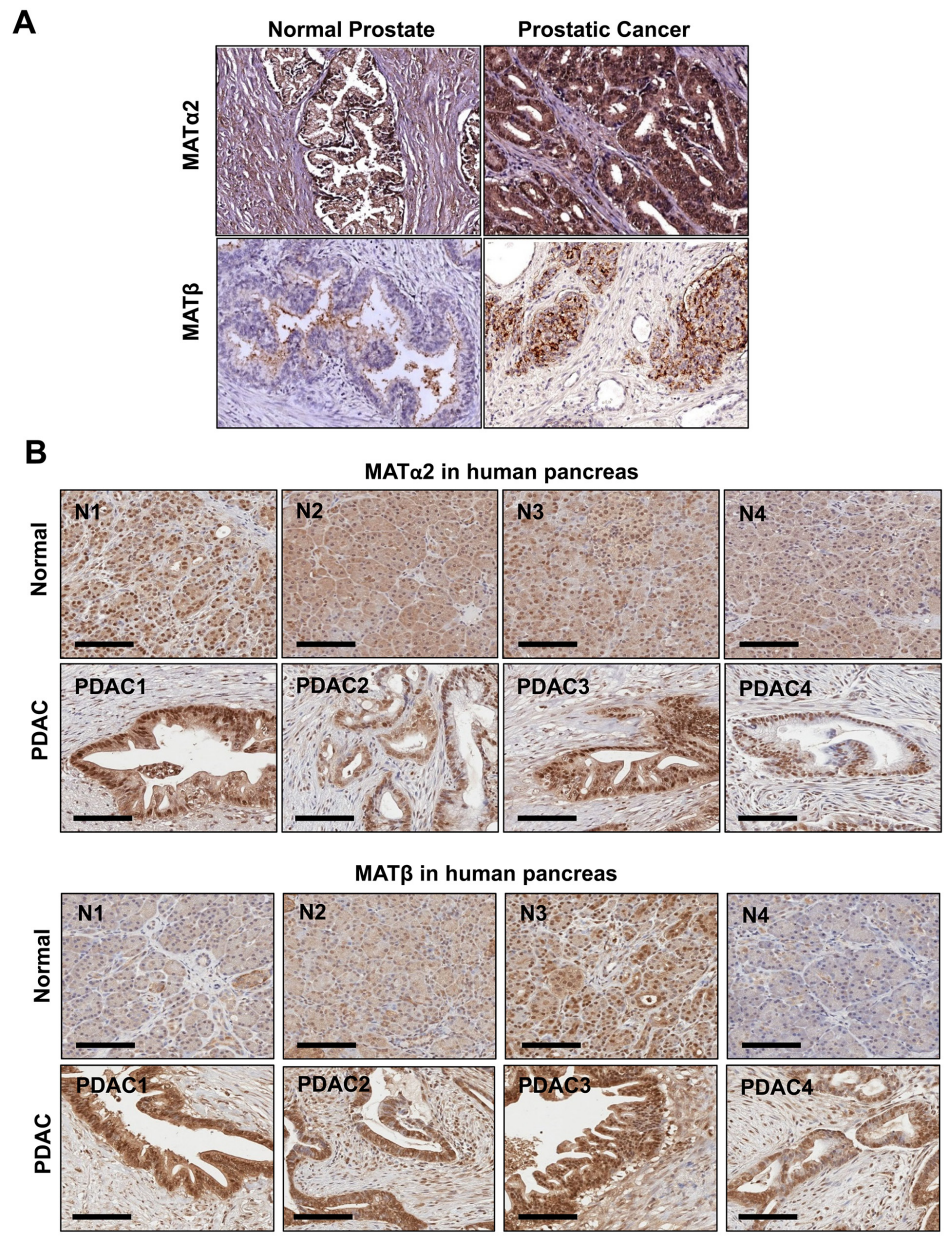
MAT2A and MAT2B expression is upregulated in human prostate and pancreatic cancers **(A)** Representative IHC of MATα2 and MATβ is shown from normal (n = 8) and prostate cancer patient tissues (n = 40). **(B)** IHC of MATα2 and MATβ from 4 separate normal pancreatic tissues and PDACs are shown, scale = 100 μm. Magnification x 20.

**Figure 7:**
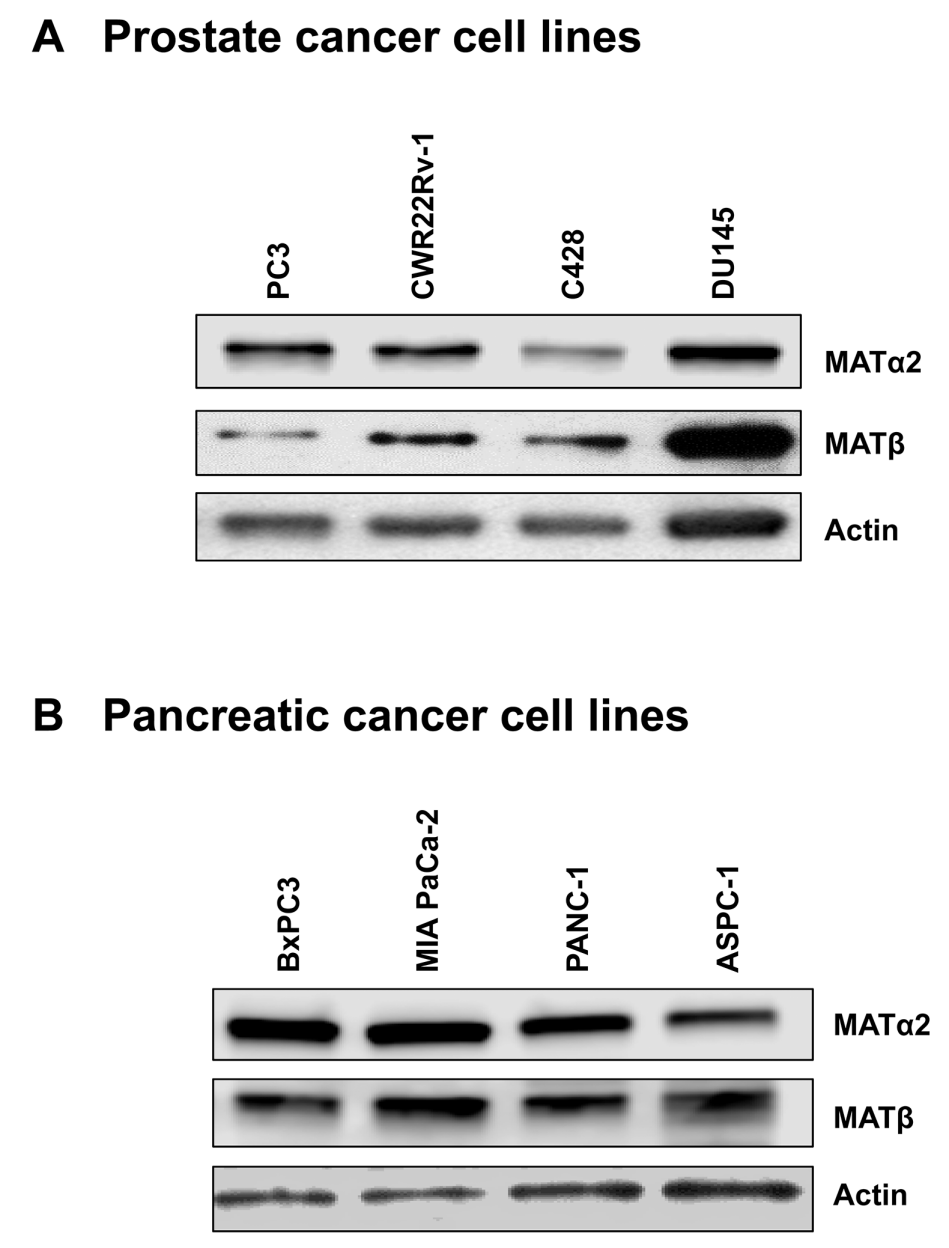
Expression of MATα2 and MATβ in prostate and pancreatic cancer cell lines Western blotting was done to compare expression level of MATα2 and MATβ from 4 prostate **(A)** and 4 pancreatic **(B)** cancer cell lines.

**Figure 8:**
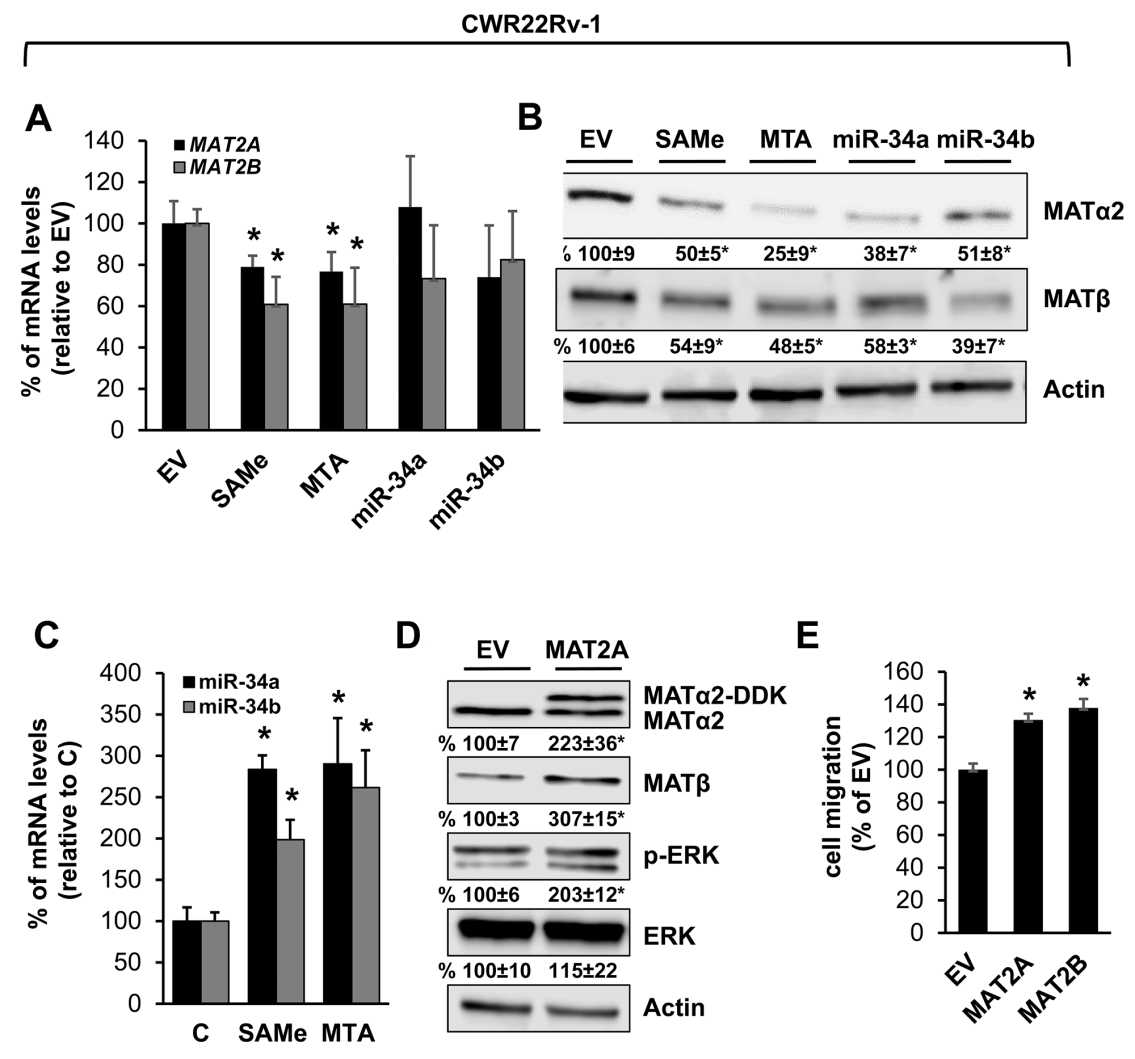
MAT2A and MAT2B expression is down-regulated by SAMe, MTA, miR-34a and miR-34b in prostate cancer cell line CWR22Rv1 cells were treated with 250 μM SAMe or MTA, or overexpression of miR-34a or miR-34b as described in Methods for 24 hours. MAT2A and MAT2B mRNA **(A)** and protein **(B)** levels were measured by real-time PCR and Western blotting. Densitometric changes are summarized below the blots. Results represent mean ± SEM from 3 to 4 experiments, *p<0.04 vs. empty vector (EV) control. **(C)** Effect of SAMe and MTA treatment (250 μM for 24 hours) on miR-34a and miR-34b expression in CWR22Rv-1 cells. Results represent mean ± SEM from 5 experiments, *p<0.05 vs. control. **(D)** CWR22Rv-1 cells were transfected with MAT2A overexpression vector in DDK tag for 24 hours and Western blotting was done for MATα2, MATβ, p-ERK, total ERK and Actin for housekeeping control. Densitometric values are summarized below the blots. Results represent mean ± SEM from 3 experiments, *p<0.02 vs. EV. **(E)** CWR22Rv-1 cells were transfected with EV, MAT2A or MAT2B overexpression vector and cell migration was measured as described in Methods. Results represent mean ± SEM from 3 experiments, *p<0.002 vs. EV control.

**Figure 9:**
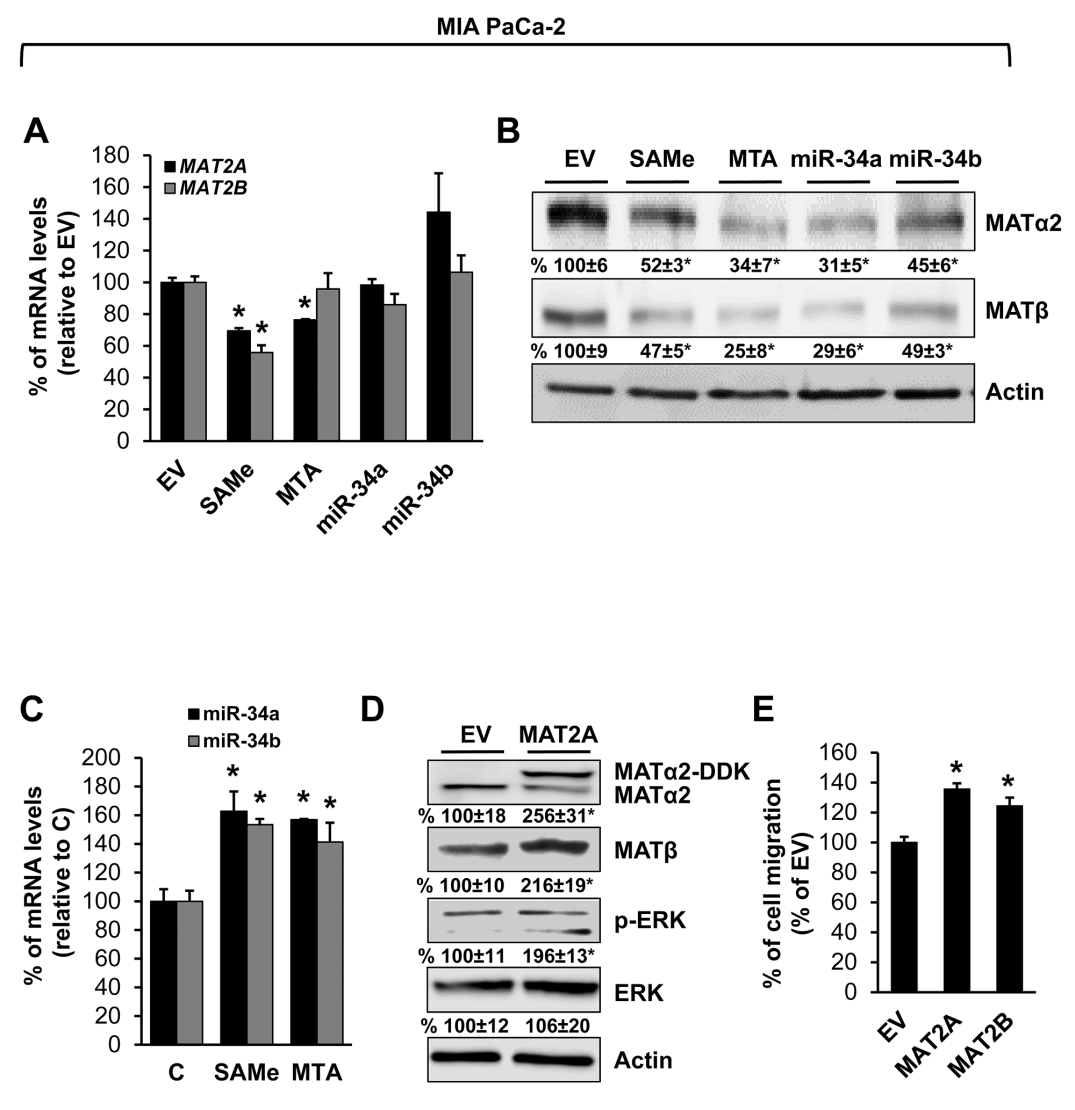
MAT2A and MAT2B expression is down-regulated by SAMe, MTA, miR-34a and miR-34b in pancreatic cancer cell line MIA PaCa-2 cells were treated with 250 μM SAMe or MTA, or overexpression of miR-34a or miR-34b as described in Methods for 24 hours. MAT2A and MAT2B mRNA **(A)** and protein **(B)** levels were measured by real-time PCR and Western blotting. Densitometric changes are summarized below the blots. Results represent mean ± SEM from 3 experiments, *p<0.02 vs. empty vector (EV) control. **(C)** Effect of SAMe and MTA treatment (250 μM for 24 hours) on miR-34a and miR-34b expression in MIA PaCa-2 cells. Results represent mean ± SEM from 4 experiments, *p<0.05 vs. control. **(D)** MIA PaCa-2 cells were transfected with MAT2A overexpression vector in DDK tag for 24 hours and Western blotting was done for MATα2, MATβ, p-ERK, total ERK and Actin for housekeeping control. Densitometric values are summarized below the blots. Results represent mean ± SEM from 3 experiments, *p<0.05 vs. EV. **(E)** MIA PaCa-2 cells were transfected with EV, MAT2A or MAT2B overexpression vector and cell migration was measured as described in Methods. Results represent mean ± SEM from 3 experiments, *p<0.03 vs. EV control.

**Figure 10:**
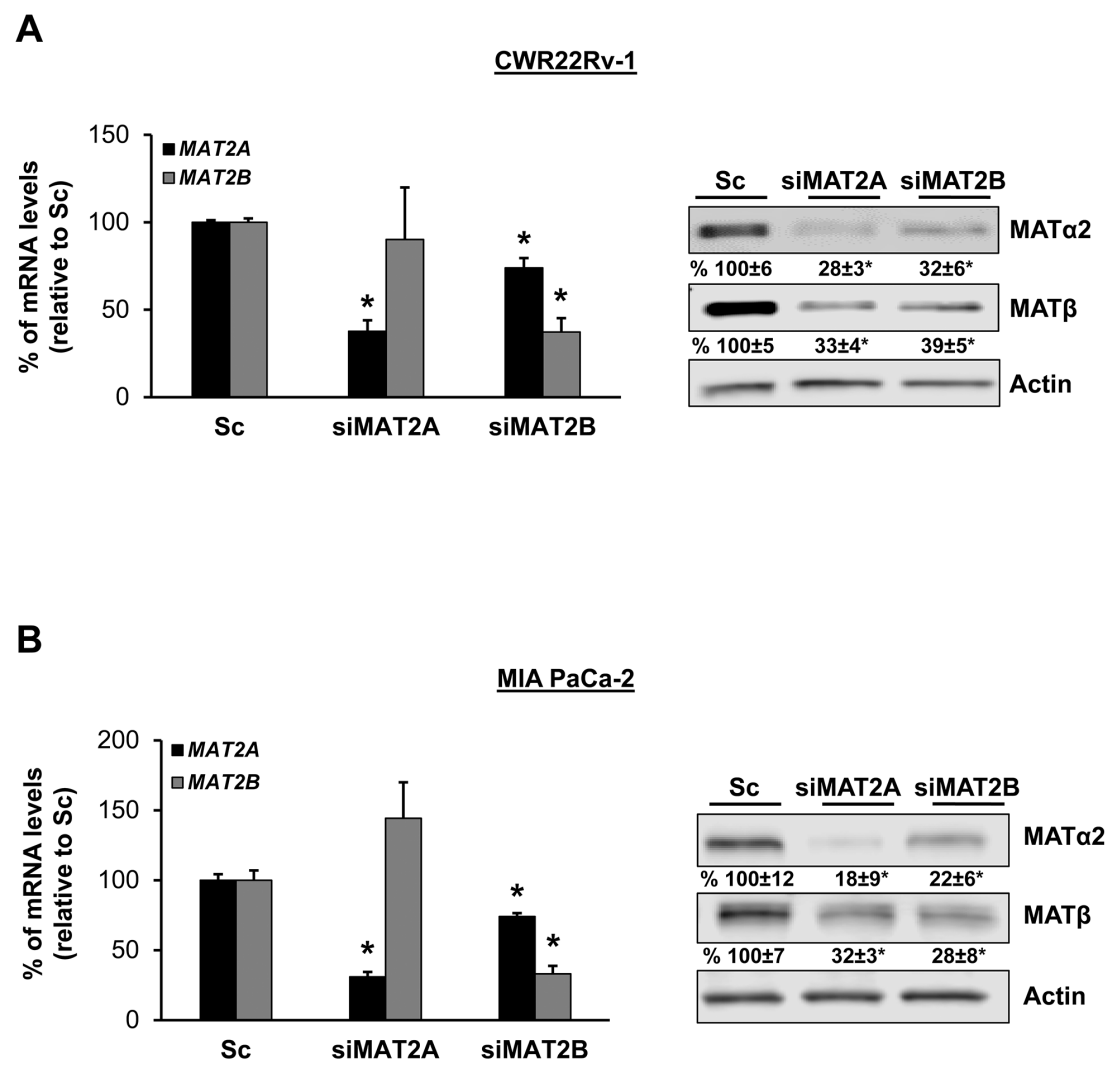
MAT2A and MAT2B proteins stabilize each other in CWR22Rv-1 and MIA PaCa-2 cells CWR22Rv-1 **(A)** and MIA PaCa-2 **(B)** cells were treated with 10 nM siRNA against *MAT2A* or *MAT2B*, or scramble siRNA control (Sc) for 48 hours and MAT2A and MAT2B expression were measured by real-time PCR and Western blotting. Densitometric changes are summarized below the blots. Results represent mean ± SEM from 3 experiments, *p<0.03 vs. Sc.

**Figure 11:**
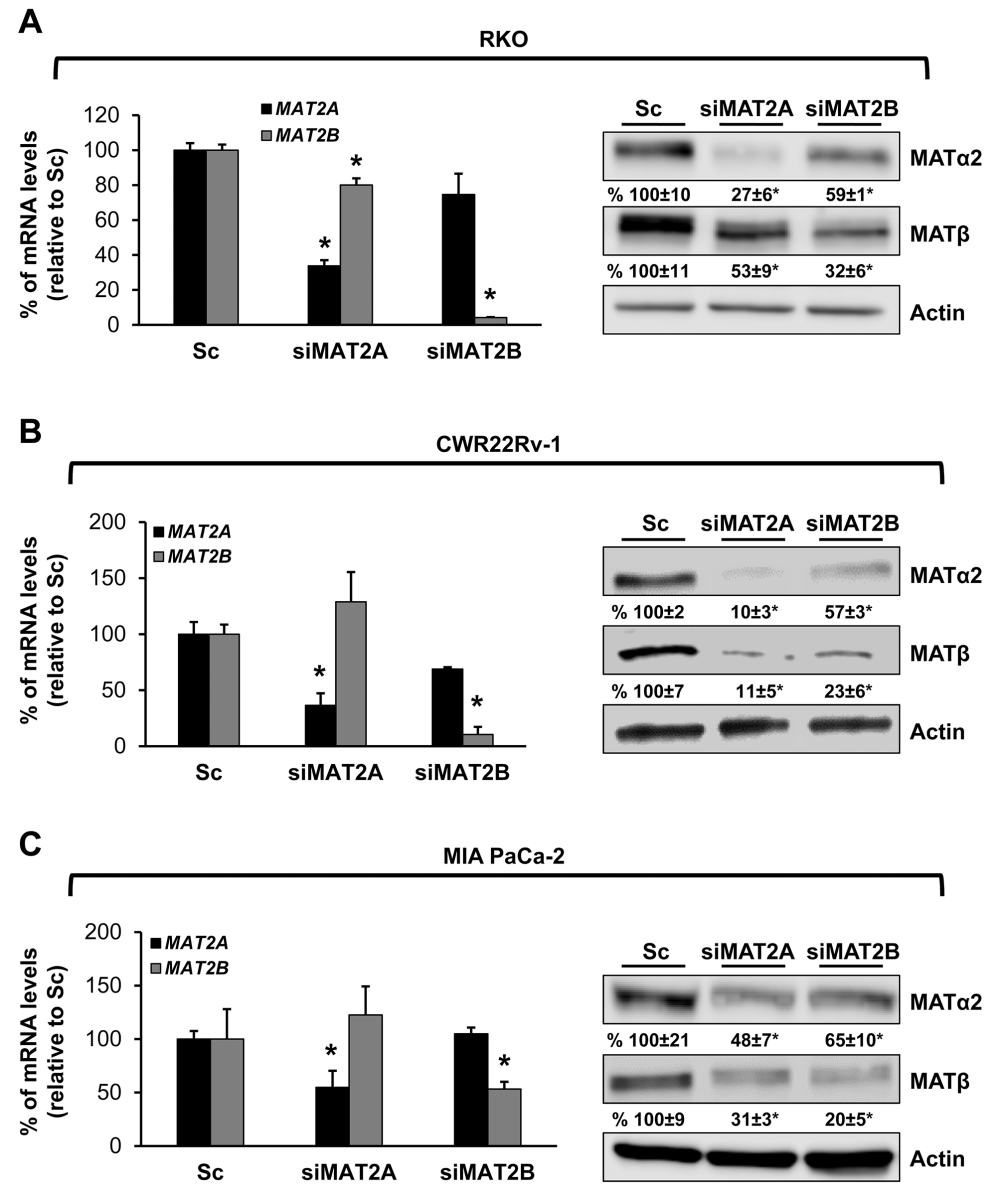
MAT2A and MAT2B proteins stabilize each other in RKO, CWR22Rv-1 and MIA PaCa-2 cells RKO **(A)**, CWR22Rv-1 **(B)** and MIA PaCa-2 **(C)** cells were treated with a second siRNA (10 nM) against *MAT2A* or *MAT2B*, or scramble siRNA control (Sc) for 48 hours and MAT2A and MAT2B expression were measured by real-time PCR and Western blotting. Densitometric changes are summarized below the blots. Results represent mean ± SEM from 3 experiments, *p<0.05 vs. Sc.

We also examined prostate and pancreatic cancers, two other human cancers where miR-34a expression is down-regulated [1, 3]. Both MATα2 and MATβ expression are higher in human prostate and pancreatic cancers (Figure 6A and 6B). We next examined whether SAMe, MTA and miR-34a/b influence the expression of these MAT genes and proteins in 22Rv-1 (human prostate cancer cell line) and MIA PaCa-2 (human pancreatic cancer cell line), which express moderate levels of these proteins as compared to multiple other prostate and pancreatic cancer cell lines (Figure 7). Treatment with SAMe, MTA, overexpression of miR-34a or miR-34b lowered the expression of MAT2A and MAT2B mainly at the protein levels in both CWR22Rv-1 (Figure 8A and 8B) and MIA PaCa-2 (Figure 9A and 9B) cells. Similar to the CRCs, SAMe and MTA treatment raised miR-34a and miR-34b expression (Figures 8C and 9C). In addition, overexpressing MAT2A raised MAT2B protein level, ERK activity (Figures 8D and 9D) and both MAT2A and MAT2B increased CWR22Rv-1 and MIA PaCa-2 cell migration when overexpressed (Figures 8E and 9E). Also similar to CRC cells, these two MAT proteins positively regulate each other’s expression as knocking down either one drastically reduced the protein level of the other (Figure 10). This dependence on each other at the protein level was verified using a second siRNA for either MAT2A or MAT2B (Figure 11).

## DISCUSSION

MAT is an essential enzyme that is required for survival as it catalyzes the formation of SAMe, the principal biological methyl donor [13]. Two MAT genes, *MAT1A* and *MAT2A*, encode for the catalytic subunit MATα1 and MATα2, which are expressed mainly in the liver and in all extrahepatic tissues, respectively. A third gene, *MAT2B*, encodes for a regulatory subunit MATβ that regulates *MAT2A*-encoded isoenzyme [13]. In addition, MATβ is part of a scaffold complex that interacts with and activates all the components of RAS-RAF-MEK-ERK signaling cascade [11, 14]. Increased MAT2A and MAT2B expression occurs in human liver and colon cancers [13] and increased MAT2A expression has also been reported in human gastric cancer [16] and tamoxifen-resistant breast cancer cells [17]. Their expression has not been reported in human prostate or pancreatic cancers. Although MAT2A and MAT2B expression are induced in parallel in liver and colon cancers, the underlying mechanism that may connect them has remained unclear.

Our current work examined whether SAMe and MTA may target the miR-34a/b-MAT2A/2B axis because of the finding from Rokavec et al. that IL-6-activated STAT3 suppressed miR-34a expression and inhibited CRC migration and invasion [5]. SAMe and MTA inhibited IL-6/STAT3 signaling and lowered tumor burden in a colitis-associated cancer model [6]. Both agents also lower MAT2A expression [7] and consensus binding sites for miR-34a and miR-34b are present in the *MAT2A* 3’-UTR. These findings raised the questions whether 1) SAMe and MTA can inhibit migration and invasion, 2) miR-34a and miR-34b can target MAT2A, and if so, 3) what is the role of MAT2A in mediating the effects of miR-34a/b.

We found both SAMe and MTA inhibited CRC cell migration and invasion, which may be in part mediated by its inductive effect on miR-34a and miR-34b. Since both agents can inhibit IL-6/STAT3 signaling [6], the suppressive effect may be mediated by this action. Silencing of miR-34b in CRC and miR-34a in Hodgkin lymphoma has been reported to be related to CpG island methylation [18, 19], which appears to contradict the role of SAMe as a methyl donor. However, the effects of exogenously delivered SAMe could be mediated by its metabolite MTA [20]. SAMe is unstable and converts to MTA spontaneously which can’t be blocked (1.3% per hour at 37°C, pH 7.0) [7]. In contrast to SAMe, MTA is highly stable, traverses the plasma membrane readily and inhibits methylation and polyamine biosynthesis [13]. Thus, if the biological effect is very rapid (so conversion to MTA is minimal), or associated with an increase in methylation, then the active molecule is SAMe. On the other hand, if the outcome is associated with a fall in methylation, then the active molecule is MTA. This is illustrated by SAMe and MTA’s inhibitory effect on lipopolysaccharide (LPS)-induced expression of proinflammatory cytokines where MTA was the active compound that blocked the LPS-mediated increase in methylated histone 3 lysine 4 (H3K4) associated with these promoters [21]. Indeed, MTA may be the active molecule in the effect of SAMe on migration, invasion and growth as it was effective at lower dosages than SAMe. We and others have also shown MTA is more potent than SAMe in its ability to inhibit liver and CRC cell growth [7, 22], exert a selective pro-apoptotic effect in both hepatocellular carcinoma and CRC (albeit by different mechanisms) [23, 24], and chemopreventive in liver and colitis-induced colon cancer [6, 25]. We suspect MTA is the mediator of SAMe on miR-34a and miR-34b expression, as MTA is a potent inhibitor of methylation [20] and previous studies have shown inhibiting DNA methylation raised the expression of both miRNAs [18, 19]. In addition, both SAMe and MTA can lower IL-6 expression and inhibit IL-6/STAT3 signaling in a colitis-associated colon cancer model and in human colon cancer cells [6]. This signaling pathway is known to suppress miR-34a [5] but whether it regulates miR-34b is unknown. Importantly, treatment with either SAMe or MTA completely prevented appearance of any tumor nodules in the metastatic CRC model. We were not able to compare miR-34a/b expression between the control and SAMe/MTA treated groups since the tumors are of human origin and no visible tumor was found in the SAMe or MTA treated groups. A SAMe pharmacokinetic study performed in humans showed plasma SAMe level increased to 127±49 μM at 1.9±0.2 hours and 211±94 μM at 1.6 ± 0.2 hours in men and women, respectively, after one intravenous infusion of 1 gm SAMe over 1.5 hours [26]. No adverse effects were reported after 5 days of intravenous SAMe administration [26]. Thus, the concentrations of SAMe that would inhibit cancer cell proliferation (65 μM) and migration (250 μM) observed in our study could be achieved in humans.

Most of the published literature shows miR-34 family members as tumor suppressors [2, 4]. All three family members are direct transcriptional targets of p53 and many of the targets of the miR-34 family members are involved in cell cycle, apoptosis, invasion and migration [2, 4]. In CRC, all three family members have been reported to be down-regulated [5, 18]. Both miR-34a and miR-34b have also been shown to behave as tumor suppressors in prostate and pancreatic cancers [1, 3, 27, 28]. Our current results add MAT2A and MAT2B to the list of their targets. Their suppressive effect on MAT2A is direct as the *MAT2A* 3’UTR has non-complementary binding sequences for miR-34a/b. Consistently, the suppressive effect lies mainly at the protein level likely due to inhibition of protein translation, a dominant mechanism for imperfect base pairing between miRNA and its target [29]. However, the effect on MAT2B is indirect. Our results support the notion that the effect on MAT2B is mediated by MAT2A, as the two encoded proteins stabilize each other. This has been previously suggested in an earlier report [30], which showed higher protein levels of MAT2A when MAT2B was co-overexpressed as compared to overexpressed alone in COS-1 cells. Here we confirmed that the two MAT proteins stabilize each other using knockdown of endogenous MAT2A and MAT2B in multiple human cancer cell lines. This helps to explain why the two proteins are often induced in parallel in multiple human cancers, which now include liver cancer, CRC, prostate and pancreas. It also helps to explain how increased MAT2A expression can induce cancer cell migration and invasion. Our results support that these are at least mediated in part via raising MAT2B’s expression, which can activate ERK [11, 14]. The finding that overexpressing miR-34a or miR-34b had minimal to no influence on the inductive effect of MAT2A/MAT2B on CRC growth, migration or invasion supports an important role of these two MAT proteins in mediating the effects of miR-34a/b on these parameters.

A recent paper from Schmidt et al [31] also reported SAMe inhibited PC-3 prostate cancer cell proliferation, migration and invasion after 144 hours of treatment. The effects on proliferation and invasion were observed with 80 μM SAMe but required 320 μM SAMe to inhibit migration. Our findings are consistent with this report and suggest SAMe and MTA could also be effective preventing prostate and pancreatic cancer metastasis. Indeed, both SAMe and MTA treatment lowered the expression of MAT2A and MAT2B, which increased migration of prostate and pancreatic cancer cells when overexpressed.

**Figure 12:**
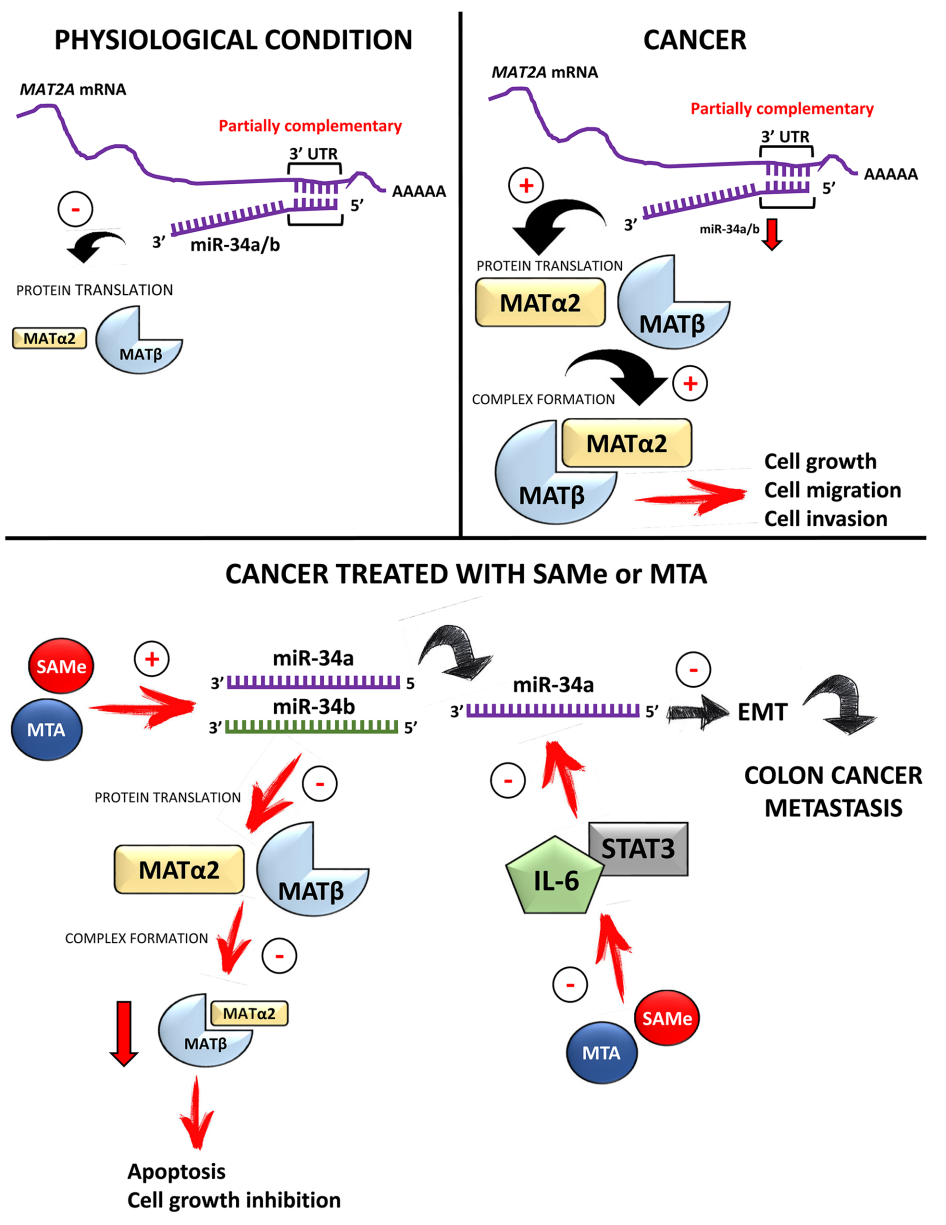
Summary diagram of miR-34a/b-MAT2A/MAT2B axis and treatment with SAMe or MTA In normal non-hepatic tissues, miR-34a and miR-34b negatively regulate MAT2A expression mainly by suppressing its protein translation. In cancer cells miRNA-34a and miR-34b are often down-regulated, releasing the inhibition on MAT2A expression. This indirectly raises MAT2B expression as the two proteins interact and stabilize each other. Higher MAT2A and MAT2B expression leads to cancer cell growth, migration and invasion. Treatment with SAMe or MTA in cancer cells increases the expression of miR-34a and miR-34b. Potential mechanisms involved include via lowering DNA methylation of miR-34a/b promoters, and inhibition of IL-6/STAT3 signaling pathway. SAMe and MTA could also suppress MAT2A and MAT2B by other mechanisms not shown. Collectively this results in lower MAT2A and MAT2B expression, increased apoptosis, decreased cell growth, migration and metastasis.

In summary, we have identified MAT2A and MAT2B as direct and indirect targets of miR-34a and miR-34b, and that the two MAT proteins are important mediators of the effect of miR-34a/b on cancer cell growth, migration and invasion. MAT2A and MAT2B proteins are induced in multiple cancers in parallel because they stabilize each other. SAMe and MTA treatment target this axis by raising the expression of miR-34a/b and suppressing that of MAT2A/MAT2B. Figure 12 summarizes the miR-34a/b-MAT2A/MAT2B axis in normal and cancer cells and the effects of SAMe/MTA treatment.

## MATERIALS AND METHODS

### Materials

SAMe, in the stable form of disulfate p-toluene sulfonate dried powder was generously provided by Gnosis SRL (Cairate, Italy) and MTA was from Sigma-Aldrich (St. Louis, MO). All other reagents were of analytical grade and obtained from commercial sources.

### Cell culture and SAMe/MTA treatment

RKO (a human colon carcinoma cell line with wild type p53), SW620 (human colon carcinoma cell line, derived from metastatic site with mutant p53), human pancreatic ductal adenocarcinoma cell lines PANC-1, MIA PaCa-2, BxPC3, and ASPC-1, and human prostate cancer cell lines CWR22Rv-1, PC3, C42b, and DU145 were obtained from the American Type Cell Collection and grown according to instructions provided. Cells were grown in 10% fetal bovine serum for 24 hours and media was changed prior to SAMe (65 μmol/L to 1 mmol/L) or MTA (65 μmol/L to 1 mmol/L) treatment for another 24 hours.

### Tissue specimens

Five pairs of pancreatic cancer and corresponding surrounding non-tumorous tissue from surgical resection were used. These were provided by the Cedars-Sinai Biobank as paraffin embedded de-identified slides. Written informed consent was obtained from each patient under Institutional Review Board of Cedars-Sinai Medical Center. The prostate benign and cancer tissues were analyzed on a tissue array (US BioMax Inc., Rockville, MD).

### Animal experiments

A total of 32 twelve-week old male J:NU mice weighing 20 to 23 g (Jackson Lab, Bar Harbor, ME) were used. While under anesthesia, intrasplenic and intrahepatic (left lobe) injection was performed using 1.5x10^6^ SW620 cells suspended in 50 μl of serum-free growth media using a 25-gauge needle delivered to each site. The peritoneum and skin were closed in a single layer with surgical suture. Three days after surgery mice received systemic delivery of 1x10^6^ SW620 cells via tail vein injection and this was repeated at day 7 and 14. On day 4 (one day after the first tail vein injection), mice were separated into four groups (n=8 per group): 1) control for SAMe, 2) SAMe, 3) control for MTA and 4) MTA. SAMe was mixed in phosphate buffered saline (PBS) and given daily at 100 mg/kg in 100 μl via gavage during the weekdays and in drinking water (amount dissolved in 6 ml, their average daily intake) on the weekend. MTA was mixed in dimethyl sulfoxide (DMSO) DMSO and given as 75 mg/kg mixed in 6ml of drinking water (final DMSO was 0.025% in water). SAMe control group received PBS gavage during the weekdays and PBS in drinking water, while MTA control group received 0.025% DMSO in drinking water. Mice were euthanized after 21 days for examination of intrahepatic tumors, peritoneum, pancreas, and lung metastasis. Tumor specimens from each treatment group were snap frozen and fixed in formalin. The tumors width and length were measured using a Vernier gauge and the volume was determined as 0.5 (width × width × length).

All procedure protocols, use, and the care of the animals were reviewed and approved by the Institutional Animal Care and Use Committee at Keck School of Medicine USC.

### RNA extraction and real-time polymerase chain reaction analysis

Total RNA isolated from cells and tissues as described [10] was subjected to reverse transcription (RT) by using M-MLV Reverse Transcriptase (Invitrogen, Carlsbad, CA). MiRNAs were extracted using the RNeasy MinElute Cleanup kit (Qiagen), then reverse transcribed into cDNA using either mercury LNATM Universal RT microRNA PCR (Exiqon, Denmark). One μl of RT product was subjected to quantitative real-time PCR analysis. The primers and TaqMan probes for *MAT2A*, *MAT2B*, miR-34a, miR-34b and Universal PCR Master Mix were purchased from ABI (Foster City, CA). *18SrRNA* and U6 snRNA were used as housekeeping genes as described [7, 12]. The delta Ct (ΔCt) obtained was used to find the relative expression of genes according to the formula: relative expression=2^-ΔΔCt^, where ΔΔCt= ΔCt of respective genes in experimental groups – ΔCt of the same genes in control group.

### RNA interference

The predesigned siRNAs targeting human *MAT2A* (siRNA1 sense sequence: 5’-GUGAGAGAGAGCUAUUAGA-3’, antisense sequence: 5’-UCUAAUAGCUCUCUCUCACUC-3’; siRNA2 sense sequence: 5’-ACACAUUGGAUAUGAUGAUTT-3’, antisense sequence: 5’-AUCAUCAUAUCCAAUGUGUTT-3’) were purchased from Sigma (St. Louis, MO), *MAT2B* (siRNA1 sense sequence: 5’-AAGAGCUCUCUAUACACUUUU-3’, antisense sequence: 5’-GCUCUCUAUAUACACUUUGUU-3’; siRNA2 sense sequence: 5’-GCUCUCUAUAUACACUUUGUU-3’, antisense sequence: 5’-AACAAAGUGUAUAGAGAGCUC-3’), and scrambled control siRNA were purchased from Ambion (Austin, TX). Cells were cultured in 6-well plate (0.4×10^6^ cells/well) and transfected using RNAiMax (5 μL/well) from Invitrogen (Carlsbad, CA) with *MAT2A*, *MAT2B* siRNA (10 nM), or scrambled control siRNA for 48 hours for mRNA or protein expression, following the manufacturer’s instructions. Scramble siRNAs did not cause any toxicity or change in the gene expression as compared to untreated controls.

### Overexpression

*MAT2A* and *MAT2B* overexpression vectors were previously described [9, 11]. MiR-34a, miR-34b and empty vectors were purchased from Origene (Rockville, MD) and GeneCopoeia (Rockville, MD), respectively. Cells were cultured in 6-well plates (0.4x10^6^ cells/well), transfected using 5 μl of JetPRIME from Polyplus (New York, NY) and 2 μg of target plasmid per well. After 4 hours, the transfection medium was changed to normal medium and the cells were cultured for an additional 20 hours various assays as indicated.

### *MAT2A* 3’UTR reporter assay

*MAT2A* 3’UTR construct was purchased from Origene (Rockville, MD). RKO cells were placed in 12-well plates the day before transfection. MiR-34a and miR-34b were overexpressed as describe above. After 24 hours transfection, *MAT2A* 3’UTR pMirTarget vector (800 ng), and a control Renilla luciferase expression vector (2.5 ng) were co-transfected into RKO cells with JetPRIME (Polyplus, New York, NY) following the manufacturer’s instructions. Luciferase assays were performed 24 hours later using the Dual Luciferase Reporter Assay System (Promega, Madison, WI) as directed by the manufacturer suggested protocol. Firefly luciferase activity was normalized to the Renilla luciferase activity.

### Western blots and immunohistochemistry (IHC)

Western blotting was performed following standard protocols (Amersham BioSciences, Piscataway, NJ), and the membranes were probed with anti-MATα2 (Novus, Littleton, CO), anti-MATβ (Genetex, Irvine, CA), p-ERK1/2 (T202, Y204) (Cell Signaling, Danver, MA) total ERK1/2 (Santa Cruz, Dallas, TX) and Actin (Sigma, St. Louis, MO) antibodies. Blots were developed using enhanced chemoluminescence. IHC for MATα2 and MATβ was performed as we described [11].

### Proliferation and apoptosis assays

Proliferation assay was measured using the Bromodeoxyuridine (BrdU) Cell Proliferation Assay Kit (CalBiochem, San Diego, CA). Apoptosis was determined by nuclei Hoechst staining as we described [8].

### Migration and invasion assays

The 2-D cell migration was measured using Radius™ 24-Well Cell Migration Assay kit from Cell Biolabs Inc. (San Diego, CA, USA) by following manufacturer’s instructions. Briefly, 0.4x10^6^ cells were seeded onto the radius of a 24-well plate and treated as indicated. After 24 hours, photographic images were acquired under an inverted microscope (EVOS XL core, Life technologies) and the migration occupied area was measured using the Image J software.

The invasion assay was assessed using the QCM™ 24-well cell invasion chamber from Millipore (Temecula, CA). Briefly, 2.5x10^5^ cells were placed onto the top insert. The bottom of the cell insert was covered with a filter containing multiple 8-μm pores and coated with a basement membrane matrix (Matrigel). Cells, in 500 μl of serum-free MEM media, were seeded in the cell insert and placed in the well, which was filled with 750 μl of MEM and supplemented with 10% fetal bovine serum. After 16 hours of plating the cells were treated as indicate for an additional 24 hours. Invaded cells on the bottom of the insert membrane were detached and subsequently lysed and detected by CyQuant GR dye.

### Statistical analysis

Data are expressed as mean±SEM. Statistical analysis was performed using ANOVA and Fisher’s test. For mRNA and protein levels, ratios of genes and proteins to respective housekeeping densitometric values were compared. Significance was defined by p<0.05.

## Abbreviations

BCL-2: B-Cell CLL/lymphoma 2
BrdU: 5-Bromo-2’-deoxyuridine
CRC: colorectal cancer
DMSO: dimethyl sulfoxide
IHC: immunohistochemistry
IL-6: interleukin-6
LPS: lipopolysaccharide
MAT: methionine adenosyltransferase
miRNA: microRNA
MTA: methylthioadenosine
PBS: phosphate buffered saline
PCR: polymerase chain reaction
PDAC: pancreatic ductal adenocarcinoma
RT: reverse transcription
SAMe: S-adenosylmethionine
siRNA: small interfering RNA
STAT3: Signal transducer and activator of transcription 3
