# Correction: S-adenosylmethionine and methylthioadenosine inhibit cancer metastasis by targeting MicroRNA 34a/b-methionine adenosyltransferase 2A/2B axis

**DOI:** 10.18632/oncotarget.28858

**Published:** 2026-04-24

**Authors:** Maria Lauda Tomasi, Carla Cossu, Ylenia Spissu, Andrea Floris, Minjung Ryoo, Ainhoa Iglesias-Ara, Qiang Wang, Stephen J. Pandol, Neil A. Bhowmick, Ekihiro Seki, Edwin M. Posadas, Shelly C. Lu

**Affiliations:** ^1^Division of Digestive and Liver Diseases, Department of Medicine, Cedars-Sinai Medical Center, Los Angeles, California, USA; ^2^Department of Medicine, University of Sassari, Sassari, Italy; ^3^Department of Biomedical Science, University of Cagliari, Cagliari, Italy; ^4^Department of Genetics, Faculty of Science and Technology, University of The Basque Country, Bilbao, Spain; ^5^Department of Biomedical Sciences, Cedars-Sinai Medical Center, Los Angeles, California, USA; ^6^Urologic Oncology Program, Division of Hematology and Oncology, Department of Medicine, Cedars-Sinai Medical Center, Los Angeles, California, USA; ^7^Translational Oncology Program, Samuel Oschin Comprehensive Cancer Institute, Cedars-Sinai Medical Center, Los Angeles, California, USA; ^8^Cancer Biology Program, Samuel Oschin Comprehensive Cancer Institute, Cedars-Sinai Medical Center, Los Angeles, California, USA; ^*^Shared first-authorship

**This article has been corrected:** It was identified that the H&E staining image of mice liver from the control SAMe group in Figure 5A overlaps with the H&E control MTA group image in Figure 5B.

The authors have confirmed that the control SAMe H&E liver image was erroneously used for the control MTA group due to the high similarity between the samples. A revised Figure 5 has been provided, which includes the correct H&E image for the control MTA group. This correction does not change the results or conclusions of this paper.

The corrected Figure 5, produced using the original data, is shown below.

Original article: Oncotarget. 2017; 8:78851–78869. 78851-78869. https://doi.org/10.18632/oncotarget.20234

**Figure 5:**
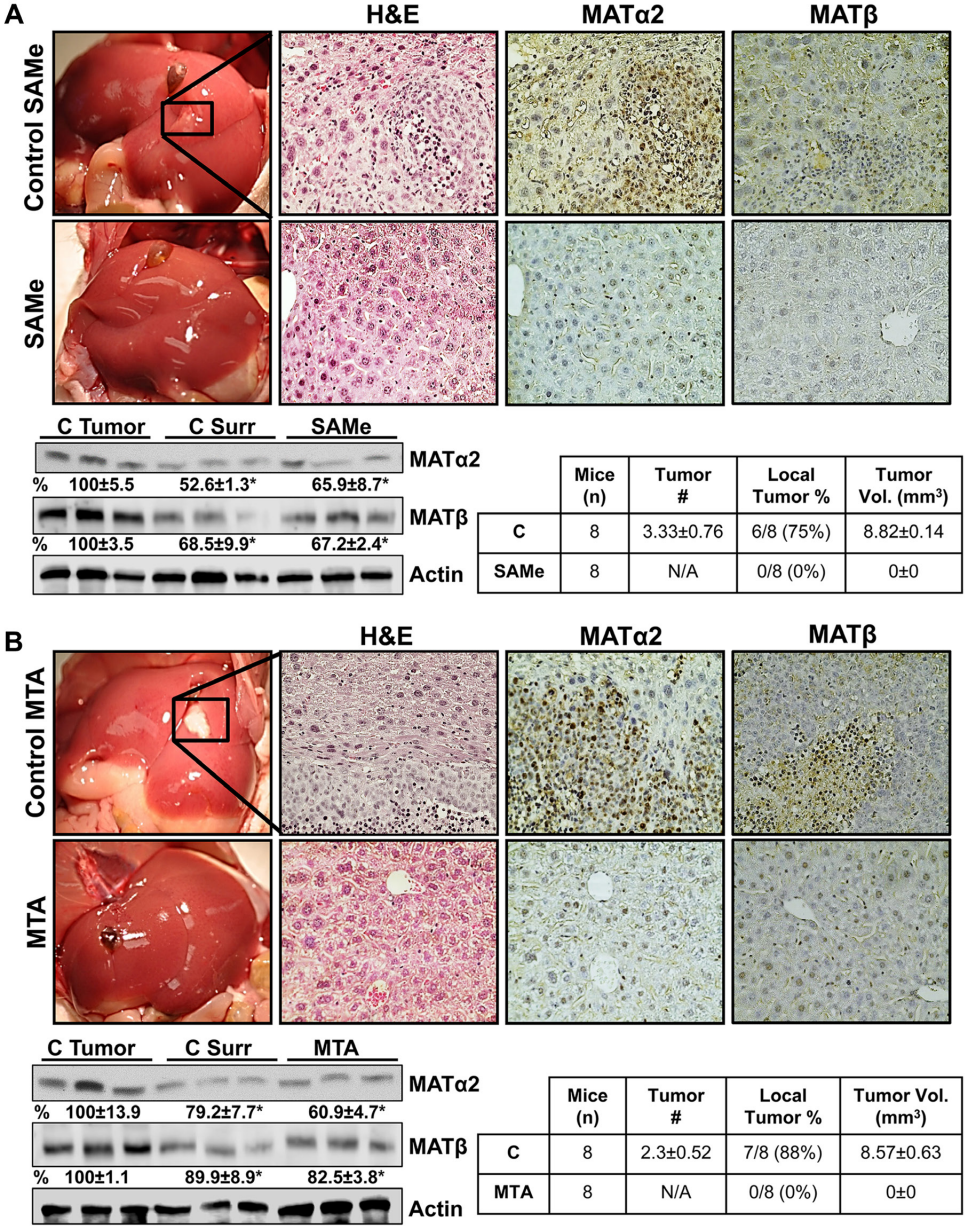
SAMe and MTA inhibit metastatic colon cancer growth in mice. A metastatic CRC model was established as described in Methods. On day 4, mice were allocated to 4 groups (*n* = 8 per group): (1) vehicle control for SAMe, (2) SAMe (100 mg/kg/day), (3) DMSO (vehicle for MTA), and (4) MTA (75 mg/kg/day). All mice were euthanized on day 21. (**A**) shows gross liver morphology, H&E and IHC for MATα2 and MATβ in the SAMe treated and control groups. (**B**) shows the same for the MTA and its control group (magnification 40x). SAMe and MTA treated groups did not have visible tumor, whereas both control groups had tumors in the majority. Some of the mice had one tumor easily visible, whereas others had multiple tumors. Tumor volume is an average of the tumors in each mouse. Representative Western blotting for MATα2 and MATβ are shown comparing their expression in the tumors, surrounding liver tissues, and SAMe or MTA treated livers from *n* = 5 per group. Densitometric values are summarized below the blots, ^*^p < 0.03 vs. respective tumor controls.

